# The WHO glove use pyramid: knowledge gaps among Belgian nurses

**DOI:** 10.1186/1753-6561-5-S6-P114

**Published:** 2011-06-29

**Authors:** D De Wandel, D Vogelaers, S Blot

**Affiliations:** 1 Faculty of Health care, University College Ghent, Ghent, Belgium; 2 Faculty of Medicine and Health Sciences, Ghent University, Ghent, Belgium; 3 Department of General Internal Medicine and Infectious Diseases, Ghent University Hospital, Ghent, Belgium

## Introduction / objectives

Appropriate glove use is a cornerstone in effective hand hygiene programs. Nurses’ knowledge regarding appropriate glove use has only rarely been studied in Belgium.

## Methods

We developed a questionnaire based on the WHO glove use pyramid. The pyramid was used as campaign material for the most recent national hand hygiene campaign (end of campaign May, 2009).Â The final questionnaire contained 36 glove use indications with four response alternatives: “no gloves indicated”, “non-sterile gloves indicated”, “sterile gloves indicated” and “I do not know”. Demographic data such as sex, age, years of nursing experience and type of ward where respondents worked, were also collected. The questionnaire was completed during class by nurses following a Bachelor-after-Bachelor’s course in the spring of 2009.

## Results

The questionnaire was filled out by exactly 100 nurses (response 100%). Maximum score was 94%, minimum 22%. The median total knowledge score (score_TOT_) was 81% (IQR 75-86). Some of the most striking gaps in knowledge were;

- 18% do not wear gloves when performing a venal puncture

- 37% wear gloves when providing basic hygienic care and 18% wear no gloves when performing genital care (as a part of hygienic care)

- 29% will manipulate vascular catheters without gloves, 24% use sterile gloves

- 58% prepare cytostatics with non-sterile gloves

The median score_TOT_ for all acute care wards was 81% (IQR 78-85), respondents providing chronic or extramural care scored 75% (IQR 71-83). This difference was statistically significant (Mann whitney U test P<0.001).

## Conclusion

We identified several knowledge gaps concerning appropriate glove use in Belgian healthcare workers. Nurses working in acute care wards scored significantly higher compared to nurses working in other wards.

## Disclosure of interest

None declared.

